# Double Retinal Tamponade for Treatment of Rhegmatogenous Retinal Detachment with Proliferative Vitreoretinopathy and Inferior Breaks

**DOI:** 10.1155/2020/6938627

**Published:** 2020-10-07

**Authors:** Mohamed Farouk Abdelkader, Shaaban Abd-Elhamid Mehany Elwan, Ahmed Shawkat Abdelhalim

**Affiliations:** Ophthalmology Department, Faculty of Medicine, Minia University, El-Minia, Egypt

## Abstract

**Purpose:**

To evaluate the efficacy and safety of the simultaneous use of short-term perfluoro-n-octane (PFO) with perfluoropropane (C3F8) gas to achieve retinal reattachment in eyes with rhegmatogenous retinal detachment (RRD) with proliferative vitreoretinopathy (PVR) grade C and multiple retinal breaks including inferior breaks.

**Design:**

This is a prospective interventional case series study. *Patients and Methods*. The study was a prospective noncomparative interventional study. It included 30 eyes of 30 patients who had RRD with PVR grade C and multiple retinal breaks including inferior tears attending the vitreoretinal unit of Minia University Hospital, Egypt. The mean age was 50.2 ± 10.63 years; 18 patients were females and 12 were males. Combined phacoemulsification and 23 G pars plana vitrectomy (PPV) with double retinal tamponade by C3F8 and PFO were done, and PFO was removed in 10–14 days. The patients were followed up for one year. The primary outcome was to achieve successful retinal reattachment, and the secondary outcomes were visual improvement and occurrence of complications.

**Results:**

Successful retinal reattachment was obtained in 28 eyes out of 30 (93.3%), and 2 eyes (6.7%) had recurrent RD. Best-corrected distance visual acuity (BCDVA) in logMAR was significantly improved from baseline 1.74 ± 0.05 to 0.93 ± 0.04, 0.82 ± 0.05, 0.80 ± 0.07, and 0.73 ± 0.055 at follow-up visits 3, 6, and 9 months and one year, respectively (*P* ≤ 0.001). There were no serious ocular complications recorded.

**Conclusions:**

The results of this study indicated that primary vitrectomy with simultaneous use of both C3F8 and short-term PFO as retinal tamponades was effective and safe in the management of complex cases of RRD with PVR grade C and inferior breaks. This trial is registered with NCT04168255.

## 1. Introduction

Surgery for the treatment of rhegmatogenous retinal detachment (RRD) with advanced proliferative vitreoretinopathy (PVR) and multiple retinal breaks including inferior breaks is a great challenge. Inferior breaks were reported to be associated with surgical failure following PPV for RRD repair. The scleral buckling versus primary vitrectomy in the RRD study (SPR group) showed that inferior detachment with breaks below the 4 and 8 o'clock positions was a significant risk factor [1]. C3F8 is a well-known intraocular gas used in vitrectomy for complicated retinal detachments, but as it is lighter than water, it provides more support to the upper quadrants of the retina than the lower retinal quadrants which means that C3F8 alone may not be sufficient to seal lower retinal breaks in cases of RRD with advanced PVR [2–4].

Perfluorocarbon liquids (PFCLs) are well known for use in vitreous surgery. They have physical properties that make them an ideal intraoperative tool in complicated vitreoretinal surgery with better efficiency and safety [5]. The moderate surface tension of PFCLs ensures that they stay cohesive after intravitreal injection. Their transparency and colorless nature ensure that their use does not affect the visualization during surgery and allow endolaser photocoagulation and their low viscosity makes them easier to inject and to remove. PFCLs are heavier than water which means that they provide more support to the lower retinal quadrants than the upper retinal quadrants [6–9].

So, the aim of this study is to evaluate the efficacy and safety of vitrectomy with the simultaneous use of short-term PFO with C3F8 to achieve retinal reattachment in eyes with RRD with PVR grade C and multiple retinal breaks including inferior breaks.

## 2. Patients and Methods

This was a prospective interventional case series study which included 30 eyes of 30 patients with RRD, PVR grade C, and multiple retinal breaks including inferior breaks (18 females and 12 males) in the period from January 2017 to December 2019 at the vitreoretinal unit of the Ophthalmology Department of Minia University Hospital, Egypt, which is a tertiary referral center for difficult cases of retinal surgery in Minia Governorate and Upper Egypt. Approval of the study was provided by the Faculty of Medicine, Research Ethical Committee, Minia University, and it was in agreement with the tenets of the Declaration of Helsinki. All patients signed an informed consent explaining the risks and benefits of the operation. All patients were followed up for 12 months.

### 2.1. Inclusion Criteria

All patients had RRD with PVR grade C and multiple retinal breaks including inferior breaks between 4 and 8 o'clock hours of the retina. All patients were medically fit for the operation and completed all the required follow-up visits.

### 2.2. Exclusion Criteria

Patients with RRD without inferior breaks, other causes of retinal detachment (tractional or exudative), penetrating ocular trauma, other ocular comorbidities which may affect visual recovery such as retinal vascular disorders, maculopathies, uveitis, glaucoma, corneal opacities, and optic nerve diseases, visual acuity less than hand motion with a good perception of light, and previous retinal reattachment surgery or intravitreal injections were excluded. Also, patients who did not complete all the required follow-up visits were excluded.

### 2.3. Preoperative Examinations

History taking included age, sex, laterality, duration, systemic diseases, and medications. Complete ophthalmological examination was performed including visual acuity assessment, intraocular pressure (IOP) measurement, slit-lamp examination of the anterior segment, dilated fundus examination using indirect ophthalmoscope as well as with the auxiliary lenses and slit-lamp aid, retinal chart drawing, biometry, and ultrasonography if needed. In biometry, the axial length was measured by combined applanation vector A/B-scan, and the SRK/T formula was used to calculate IOL power [10].

### 2.4. Surgical Technique

All surgeries were done under peribulbar anesthesia. First, phacoemulsification was performed through the clear corneal incision with implantation of hydrophobic acrylic IOL in the capsular bag and closure of the wound by a 10/0 nylon suture. This was followed by 23 G pars plana vitrectomy. Core vitrectomy was done, and triamcinolone acetonide was injected to ensure complete posterior vitreous detachment. All epiretinal and subretinal membranes were removed. ILM was peeled in all eyes using brilliant blue G (DORC International). PFO (Auro octane, Aurolab, India) was injected slowly over the optic disc to flatten the posterior retina, and base vitrectomy was performed helped by the aid of scleral depression. More PFO was injected to drain subretinal fluid through the original retinal breaks. All retinal breaks were surrounded by 2–3 confluent rows of diode laser endophotocoagulation. Laser endophotocoagulation was applied to 360 degrees of the retinal periphery. Then, air-fluid exchange was performed to aspirate PFO leaving a part of it up to the equator enough to tamponade the lower half of the retina, and 12% C3F8 (Alchimia Srl, Italy) was injected into the eye so that the eye was left with both PFO and C3F8. No scleral buckles were applied and no relaxing retinotomies were performed. The sclerotomies were sutured by 7/0 Vicryl and subconjunctival injection of triamcinolone acetonide was given at the end of surgery. Between 10 and 14 days, PFO was completely aspirated and replaced with 12% C3F8. Any PFO in the anterior chamber, if present, was aspirated.

### 2.5. Postoperative Management

All patients were given topical prednisolone acetate 1% eye drops 4 times daily and tapered through 6 weeks, cyclopentolate 0.5% eye drops 3 times daily, moxifloxacin 0.5% eye drops 3 times daily for 2 weeks, and an ointment of tobramycin 0.3% and dexamethasone 0.1% at night for 4 weeks. Postoperatively, patients were advised to adopt an upright or semisitting posture for most of the day and they were instructed to avoid facedown position until PFO was removed. Follow-up visits were advised next postoperative day, 3^rd^ day, and 1 week, and after the second procedure of PFO removal, patients were examined in the next postoperative day, one week, two weeks, one month, and then every three months for 1 year.

All patients underwent full ophthalmologic examination including best BCDVA which was converted to logMAR, IOP measurement, anterior segment slit-lamp examination, and dilated fundus examination. Baseline results and that of 3^rd^, 6^th^, and 9^th^ months and 1 year were included in the statistical analysis. The primary outcome of the study was to achieve successful retinal reattachment, and the secondary outcomes were the improvement in the visual acuity and the incidence of complications.

### 2.6. Statistical Analysis

Statistical analysis was performed with SPSS 20. Data were expressed as mean ± standard deviation (SD). Changes in the mean BCDVA were compared for each follow-up visit with baseline using paired *t*-test, and graph construction was done by using GraphPad Prism 5 program. *P* value < 0.05 was considered statistically significant.

## 3. Results

### 3.1. Preoperative Data

As shown in Table 1, the number of patients was 30 with 30 eyes included in the study. The mean age was 50.2 ± 10.63 (range, 18–64 years). Eighteen patients (60%) were females and 12 (40%) were males. Cataract was present in 26 eyes (87%), but none of them had dense cataract enough to prevent adequate examination of the retina. All eyes had myopia of ≥6 diopters. History of onset of retinal detachment ranged from 4 to 7 weeks. Total retinal detachment with macula-off was present in all eyes (100%) with PVR grade C. Giant retinal breaks involving the lower retina were present in 2 eyes (6.66%). Both of them were more than 180° but less than 270° and involved the temporal and lower parts of the retina. All eyes had ≥2 retinal breaks (range, 2–6) with at least one lower retinal tear between 4 and 8 o'clock positions. The mean duration of postoperative PFO stay in the vitreous was 12 days (range, 10–14 days). The mean baseline BCDVA in logMAR was 1.73 ± 0.05 (range, 1.3–2.2).

### 3.2. Rate of Successful and Failed Retinal Reattachment in Absence of Retinal Tamponades

All eye had retinal reattachment at the end of surgery. In 28 eyes (93.3%), successful retinal reattachment was maintained throughout the follow-up period of 1 year. Recurrent retinal detachment was observed in 2 eyes in the 3^rd^ month due to inferior PVR and was reoperated upon with lower 180° relaxing retinotomy due to retinal shortening and silicone oil (5000 centistoke) injection, and the retina was successfully attached.

### 3.3. Visual Acuity Change

**Figure 1:**
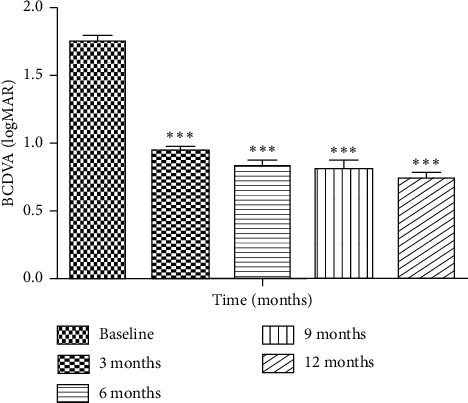
BCDVA change overtime.

The BCDVA in logMAR was significantly improved from baseline 1.74 ± 0.05 to 0.93 ± 0.04, 0.82 ± 0.05, 80 ± 0.07, and 0.73 ± 0.055 at postoperative follow-up visits 3, 6, and 9 months and one year, respectively (*P* ≤ 0.001). No patient lost his preoperative level of vision as the 2 eyes with recurrent RD retained their preoperative visual acuity (Table 2 and Figure 1).

### 3.4. Postoperative Complications

Iritis with fibrinous exudates over the IOL was found in 4 eyes (13.3%) in the first postoperative week, and it was controlled by increasing the frequency of topical steroids, and all inflamed eyes became quite after PFO removal. Elevated IOP above 21 mmHg but less than 30 mmHg was found in 5 eyes (16.6%) in the first 2 weeks and was controlled by medical treatment by a combination of beta-blocker and carbonic anhydrase inhibitor eye drops, and none of these eyes needed antiglaucoma surgery. Mild epiretinal membrane over the macula with maintained foveal contour and central retinal thickness of <300 *µ*m by OCT occurred in 2 eyes (6.6%) in the 6^th^ month which did not need surgery. Posterior capsular opacification was found in 17 eyes (56.6%) between 6 and 9 months and was treated successfully with YAG laser posterior capsulotomy. PFO was found in the anterior chamber in 3 eyes (10%) and was aspirated at the time of PFO removal. No residual PFO under the macula occurred in any eye. There were no serious ocular complications recorded in the study such as hypotony, optic nerve atrophy, suprachoroidal hemorrhage, or endophthalmitis (Table 3).

## 4. Discussion

Management of RRD with PVR grade C and multiple breaks including inferior breaks is challenging because the ordinary silicone oil and gases are lighter than water and provide an inadequate tamponading effect to the lower retina allowing fluid to pass through retinal breaks preventing the development of good laser retinopexy. Also, as the surface of the lower retinal periphery is not efficiently covered by the silicon oil or gas, inflammatory mediators and proliferative cells accumulate there leading to the formation of epiretinal membranes and recurrence of inferior retinal detachment [11].

As the presence of inferior breaks was a surgical challenge, some surgeons combined PPV with scleral buckling to produce an inferior indent because intraocular tamponade with gas or silicone oil could not provide direct support to the inferior retina; however, scleral buckling during PPV was not free of risks [12]. Some studied the management of inferior breaks during PPV without scleral buckling and reported that strict postoperative prone positioning was essential to tamponade inferior breaks when gas or silicone oil was used. This positioning was difficult and uncomfortable for many patients. Posturing periods in the literature varied from 8 to 12 days [13].

Heavy silicone oils can be used for cases of retinal detachment with inferior breaks, but they have been reported to have side effects such as intraocular inflammation, oil emulsification, increased IOP, and recurrence of upper retinal detachment. PFCLs as retinal tamponade have the advantages of having a higher tamponading effect due to their high specific gravity, and they are easier to remove than silicone oil due to their low viscosity [14–17].

In this study, combined phacovitrectomy was performed with extensive base vitrectomy, removal of all epiretinal and subretinal membranes, ILM peeling, and application of laser endophotocoagulation around all retinal breaks and to the 360 degrees of the retinal periphery. At the end of surgery, two types of retinal tamponades were used at the same time in the form of short-term PFO to seal lower retinal breaks and 12% C3F8 to seal upper retinal breaks. Both retinal tamponades have greater surface tension than the ordinary silicon oil preventing fluid escape through retinal breaks till laser retinopexy created permanent chorioretinal scar around retinal breaks, increasing the success rate of retinal reattachment. We used PFO to support the lower retina as several studies showed that PFO was safe to the retina when used as a short-term vitreous substitute [18–21].

Sigler et al. used PFO for 2–3 weeks in 44 eyes with recurrent inferior retinal detachment with PVR and reported retinal reattachment after PFO removal of 86% (38 eyes) and redetachment occurred in 6 eyes (4 eyes redetached due to inferior PVR and 2 eyes redetached due to a new superior retinal break). They hypothesized that the heavy inferior PFO created superior vitreous base countertraction causing upper retinal breaks. In our study, retinal reattachment was achieved in 93.3% of eyes which was higher than that reported by Sigler et al., and also, we had no case of recurrent superior retinal detachment in our study because we used C3F8 simultaneously with PFO to support upper retina and we did 360 degrees of laser endophotocoagulation [22].

In our study, high IOP was found in 5 eyes (16.7%) and was controlled with medical treatment without the need for glaucoma surgery while Sigler et al. [22] reported high IOP in 36% of eyes with the need for glaucoma surgery in 5% of eyes which may be due to the larger amount (complete fill of PFO) and the longer duration of stay of PFO (mean of 18.3 days with a range of 2–3 weeks), presence of PFO in the anterior chamber in 32%, and intravitreal injection of 4 mg of triamcinolone acetonide at the end of surgery.

In our study, postoperative inflammation with fibrinous exudate over the IOL occurred in 4 eyes (13.3%) which was less than that reported by Sigler et al., (32%) who used a larger amount of PFO (complete fill of PFO) for longer periods of time (mean of 18.3 days with a range of 2–3 weeks) [22].

Rofail and Lee [23] retrospectively reviewed 16 eyes with giant retinal breaks without significant PVR in which PFCL was used as retinal tamponade for a mean of 16.4 days (range 6–50 days) and reported successful retinal reattachment in 93.7% which was in agreement with our results. However, they reported that visual improvement occurred in 11 (68.8%) eyes, remained the same in 2 (12.5%) eyes, and got worse in 3 (18.6%) eyes. In our study, all eyes with retinal reattachment showed improvement in visual acuity (93.3%) because the simultaneous use of C3F8 allowed the use of a small amount of PFO for a short period of time which decreased retinal toxicity.

Rush et al. [24] retrospectively reviewed charts of 39 eyes (33 of 39 eyes showed ≥ 4 retinal breaks, with 31 of 39 eyes having at least 1 inferior break, 10 of 39 eyes having giant retinal tears, and 12 of 39 eyes showing PVR) treated by vitrectomy with a complete fill of PFO as a retinal tamponade for 7–17 days which was replaced by SF6 in 4 eyes and the other eyes with C3F8 and silicone oil. They reported anatomical retinal reattachment in 92.4% with visual improvement which was comparable to our results, but only 12 eyes of their series had PVR, while in our study all eyes had PVR of grade C. Also, in Rush et al.'s study, scleral buckling was used in 10% of eyes with inferior breaks, but in our study, no scleral buckles were used. Rush et al. reported inflammation in 8 of 39 eyes (20.6%) which was more than that reported in ours (13.3%) and IOP > 21 mmHg in 35.9% of their eyes versus 16.6% in ours because the simultaneous use of C3F8 allowed us to use a small amount of PFO (fill up to 50% of the vitreous cavity in our study versus up to complete fill of PFO in their study).

Due to the high incidence of cataract in cases of vitrectomy with PFO retention in vitreous cavity reported by previous researchers [25, 26], we combined phacoemulsification with vitrectomy even though not all eyes had cataract which decreased the number of surgeries needed for each patient. Another advantage of combining phacoemulsification with vitrectomy was the ability to perform adequate base vitrectomy, increasing the incidence of retinal reattachment.

Since there is a high incidence of ERM formation in cases of RRD with severe PVR [27, 28], we peeled the ILM in all eyes which led to a decreased incidence of postoperative ERM formation (2 eyes, 6.6%). The ERM in these 2 eyes was mild with maintained foveal contour and central retinal thickness of <300 *µ*m by OCT.

Postoperative upright or semisitting postures for most of the day ensured that the upper parts of the retina were supported by C3F8 and the lower parts of the retina were supported by PFO. Also, this postoperative posture helped to decrease the time of contact between lower PFO and fovea, decreasing any possible foveal adverse effects. An important advantage of this maneuver was that patients had not to adopt a facedown position, a position which was difficult for many patients to comply with.

C3F8 gas had an efficient tamponade effect on the upper retina and it needed no operation to remove it. PFO had a better tamponading effect on the lower retina than gases and silicone oil because of its higher specific gravity (1.76), and at the same time, it was easy to remove because of its lower viscosity. The simultaneous use of both C3F8 and PFO allowed us to use smaller amounts of PFO (up to 50% fill of vitreous cavity), leading to decreased postoperative inflammation and glaucoma and increased visual improvement.

In this study, we removed PFO in 10–14 days because it was reported that laser retinopexy formed adequate chorioretinal scars within 2 weeks [29]. During the procedure of PFO removal, we found that the retinal breaks were surrounded by chorioretinal scars as identified by the presence of pigmentation around retinal breaks.

Chhablani et al. reported a sandwich technique using a combination of 14% C3F8 and silicone oil (1000 centistokes) for inferior retinal detachment. They reported that this sandwich technique achieved complete attachment of the retina and reduced the risk of inferior retinal redetachment by adequately tamponading the inferior retina. However, they had only 4 eyes with a limited follow-up (1–6 months) [30].

To the best of our knowledge, we are the first to use double retinal tamponade by PFO and C3F8 in these difficult cases of RRD with PVR grade C and multiple retinal breaks including inferior tears in the form of 12% C3F8 to tamponade upper retina and PFO to tamponade lower retina. In this series of cases with phacovitrectomy and simultaneous use of both C3F8 and PFO as retinal tamponades, we had retinal reattachment in 93.3% of eyes and the visual acuity was improved from logMAR of preoperative visual acuity (1.74 ± 0.05) to the logMAR of postoperative visual acuity at one year (0.73 ± 0.055), which was a statistically significant visual improvement.

## 5. Conclusions

The results of this study indicated that primary phacovitrectomy with the simultaneous use of both C3F8 and short-term PFO as retinal tamponades was effective and safe with mild complications and reduced number of surgeries in cases of RRD due to multiple and inferior breaks with PVR grade C. More studies with a larger number of patients, control group, and longer follow-up periods are needed to further evaluate the efficacy and safety of this technique.

## Figures and Tables

**Table 1:** Preoperative data.

Description	Values
Number	30
Age in years, mean ± SD Range in years	50.2 ± 10.63 (18–64)
Sex (M/F), %	(12/18) (40/60)
History of onset of RD	4–7 weeks
*Lens*	
Clear	4 (13%)
Partial opacity	26 (87%)
Total opacity	0
*Retinal detachment*	
(i) Total (*n*, %)	30 (100%)
(ii) Subtotal (*n*, %)	0
*PVR grade*	
Grade C (*n*, %)	30 (100%)
*Site of retinal tears*	
(i) Lower (*n*, %)	30 (100%)
(ii) Upper tears (*n*, %)	19 (63.33%)
(iii) Macular hole	0
(iv) Giant retinal tear	2 (6.66%)
Baseline BCDVA in logMAR	1.73 ± 0.05 (1.3–2.2)

**Table 2:** BCDVA change overtime.

	Baseline	3 months	6 months	9 months	12 months
BCDVA (logMAR)	1.74 ± 0.05	0.93 ± 0.04	0.82 ± 0.05	0.80 ± 0.07	0.73 ± 0.055
*P* value	≤0.001	≤0.001	≤0.001	≤0.001	≤0.001

**Table 3:** Postoperative complications.

	Frequency	Percentage
Iritis	4	13.3
Elevated IOP < 30 mm Hg	5	16.66
Mild epiretinal membrane	2	6.66
Posterior capsular opacification	17	56.6
PFO in anterior chamber	3	10
Residual PFO under the macula	0	00

## Data Availability

The data used and/or analyzed during the current study are available from the corresponding author on reasonable request.
